# Cold exposure enhances fat utilization but not non-esterified fatty acids, glycerol or catecholamines availability during submaximal walking and running

**DOI:** 10.3389/fphys.2013.00099

**Published:** 2013-05-10

**Authors:** Dominique D. Gagnon, Hannu Rintamäki, Sheila S. Gagnon, Stephen S. Cheung, Karl-Heinz Herzig, Katja Porvari, Heikki Kyröläinen

**Affiliations:** ^1^Department of Biology of Physical Activity, University of JyväskyläJyväskylä, Finland; ^2^Department of Physiology, Institute of Biomedicine, University of OuluOulu, Finland; ^3^Finnish Institute of Occupational HealthOulu, Finland; ^4^Department of Health and Rehabilitation Sciences, School of Physical Therapy, University of Western OntarioLondon, ON, Canada; ^5^Department of Kinesiology, Brock UniversitySt. Catharines, ON, Canada; ^6^Department of Psychiatry, Kuopio University HospitalKuopio, Finland; ^7^Department of Forensic Medicine, University of OuluOulu, Finland

**Keywords:** exercise, thermal responses, energy metabolism, glucose, fat

## Abstract

Cold exposure modulates the use of carbohydrates (CHOs) and fat during exercise. This phenomenon has mostly been observed in controlled cycling studies, but not during walking and running when core temperature and oxygen consumption are controlled, as both may alter energy metabolism. This study aimed at examining energy substrate availability and utilization during walking and running in the cold when core temperature and oxygen consumption are maintained. Ten lightly clothed male subjects walked or ran for 60-min, at 50% and 70% of maximal oxygen consumption, respectively, in a climatic chamber set at 0°C or 22°C. Thermal, cardiovascular, and oxidative responses were measured every 15-min during exercise. Blood samples for serum non-esterified fatty acids (NEFAs), glycerol, glucose, beta-hydroxybutyrate (BHB), plasma catecholamines, and serum lipids were collected immediately prior, and at 30- and 60-min of exercise. Skin temperature strongly decreased while core temperature did not change during cold trials. Heart rate (HR) was also lower in cold trials. A rise in fat utilization in the cold was seen through lower respiratory quotient (RQ) (−0.03 ± 0.02), greater fat oxidation (+0.14 ± 0.13 g · min^−1^) and contribution of fat to total energy expenditure (+1.62 ± 1.99 kcal · min^−1^). No differences from cold exposure were observed in blood parameters. During submaximal walking and running, a greater reliance on derived fat sources occurs in the cold, despite the absence of concurrent alterations in NEFAs, glycerol, or catecholamine concentrations. This disparity may suggest a greater reliance on intra-muscular energy sources such as triglycerides during both walking and running.

## Introduction

Studies examining energy metabolism during exercise in cold environments have demonstrated inconsistent findings in substrate availability and utilization between fat and carbohydrate (CHO) sources (Hurley and Haymes, 1982; Timmons et al., 1985; Galloway and Maughan, 1997; Layden et al., 2002). The complexity of the bioenergetic response during exercise stems from an interaction of a wide range of variables, such as the severity of climatic and seasonal conditions (i.e., temperature, wind, and humidity) (Weller et al., 1997a,b), exercise intensity and duration, experimental protocols, and inter-individual variability (i.e., fitness level, anthropometric characteristics, etc.) in thermal responses (Xu et al., 2005).

Walking and running are the two most commonly used methods of exercise even in cold environments. Weller et al. (1997a,b) examined the physiological responses to prolonged (i.e., 6 h) intermittent low- (5 km · h^−1^, 0% incline) and high-intensity (6 km · h^−1^, 10% incline) walking in cold (5°C) and neutral (15°C) environmental conditions demonstrating that low-intensity walking in the cold increased CHO oxidation, venous glucose and lactate concentrations, and oxygen consumption ($\dot{V}O_{2}$) compared to thermoneutral conditions. The authors concluded that the difference in $\dot{V}O_{2}$ was due to the added shivering thermogenesis, attempting to compensate for the increased heat loss in the cold. The likely presence of muscle cooling may also have led to an increase in $\dot{V}O_{2}$, as reduced mechanical efficiency requires more energy to perform the same workload (Oksa et al., 2002). These results can only partially define fuel selection changes in the cold since $\dot{V}O_{2}$ was not standardized between conditions. Importantly, these changes were not observed between cold and neutral conditions in the studies of Weller et al. (1997a,b) when heat production from exercise intensity was sufficient to maintain thermoregulatory functions and $\dot{V}O_{2}$ at a similar level. Another study has also observed greater CHO utilization, during cycling, in cold environments along with increased $\dot{V}O_{2}$ (Galloway and Maughan, 1997). The possible increase in $\dot{V}O_{2}$ from the presence of shivering and muscle cooling may modulate energy metabolism, and more specifically oxidative pathways (Tipton et al., 1997; Haman et al., 2002; Oksa et al., 2002).

Most cold exercise studies have been performed during cycling, whereas physiological responses to cold during walking and running are not well known. Interestingly, energy utilization during treadmill exercise in temperate environments relies more heavily on fat sources compared to cycling (Snyder et al., 1993; Achten et al., 2003; Basset and Boulay, 2003). Differences explaining these findings include a greater muscle mass activation working at a lower intensity (Hermansen and Saltin, 1969) and blood flow (Matsui et al., 1978). The use of a greater muscle mass has been shown to increase catecholamine release (Savard et al., 1989; Kjaer et al., 1991). In addition to this increase, there is generally greater sympatho-adrenal secretion with cold exposure as well, strongly influencing lipolytic activity in adipose tissue beds through β-adrenergic receptors. This phenomenon has been well documented by Frank et al. (1997) and Castellani et al. (1998, 1999). However, Febbraio et al. (1996), suggested that there is a blunted catecholamine response during cycling when cooling reduces the rise in body temperature. Activation of greater muscle mass, through walking and running, could result in more evenly distributed heat production and circulation and provide a more suitable environment for the metabolic impacts of catecholamine release and transport.

Substrate availability and utilization during exercise have previously been believed to be unaffected unless a decrease in core temperature was observed (Hurley and Haymes, 1982), which has been associated with an increase in central drive in lipolysis and greater use of lipids (Clavert et al., 1972). However, a study by Layden et al. (2002) concluded that in normothermic subjects, reduced fat oxidation during submaximal cycling in the cold was linked to reduced free fatty acid availability, possibly due to lower blood flow in subcutaneous adipose tissue. Their results also demonstrated reduced levels of blood glucose, possibly from increased utilization in the cold. The significant muscle mass activation and blood flow during walking and running could therefore minimize the impairment of free fatty acids (FFA) availability, and possibly prevent any of its decay, depending on cold exposure severity and body heat production.

The aim of the present study was to investigate the changes in energy availability and utilization during submaximal walking and running, without a reduction in core temperature and maintained relative oxygen uptake, in a cold environment. We hypothesized that the energetic response from walking and running in the cold compared to a temperate environment would either remain unchanged or slightly increase availability and the use of fat as an energy source.

## Methods

### Participants

Ten males, moderately active and not cold acclimatized, volunteered for the study. Each participant provided informed and written consent and was screened with a PAR-Q (Physical Activity Readiness) questionnaire and for cardiovascular and respiratory conditions that could be aggravated by cold exposure. Mean (±SD) characteristics of the subjects were: age, 24.3 ± 3.0 years; height, 1.80 ± 0.10 m; body mass, 81.4 ± 10.6 kg; body surface area, 2.01 ± 0.19 m^2^; and maximal oxygen uptake ($\dot{V}O_{2\max}$), 52.3 ± 5.2 ml · kg^−1^ · min^−1^. The study was performed according to the declaration of Helsinki and was approved by the ethical committee of Central Finland Health Care District.

The subjects were required to attend one preliminary and four experimental sessions which were separated by at least 72 h and trials were performed at the same time each day to control for circadian effects. They were requested to abstain from the consumption of alcohol and caffeine, the use of tobacco and vigorous exercise for 24 h prior to each session. They were also instructed to record their dietary and fluid intake 24 h before the first experimental session, and to keep the same nutritional guidelines for each day preceding a session. They were also instructed to arrive at the laboratory between 0700 and 0800 h in a fasted state. Water was available *ad libitum* before all sessions.

### Preliminary session

During this session, height and body mass were measured and percent body fat was estimated by hydrostatic underwater weighing (Brosek et al., 1963) with the water temperature maintained at 32°C. Total body surface area (m^2^) was calculated from height (H) and weight (W) measurements as follows: *A*_*D*_ = 0.202 × W^0.425^ × H^0.725^ (Dubois and Dubois, 1916). Thereafter, the subjects performed a direct incremental running $\dot{V}O_{2\max}$ exercise test on a motorized treadmill (Tunturi T40, Accell Group, Heerenveen, The Netherlands) in a climatic chamber set at an ambient temperature of 22°C, 40% relative humidity (RH), and 0.2 m · s^−1^ wind speed.

### Experimental protocol

The protocol targeted two exercise intensities (walking and running at 50% and 70% $\dot{V}O_{2\max}$, respectively) It was established based from IREQ_min_ equations (ISO 11079, 2007) that these intensities would be enough to prevent a decrease in core temperature. Ambient temperatures of 0°C and 22°C were used at both exercise intensities (Timmons et al., 1985; Sink et al., 1989; Layden et al., 2002).

For each trial, clothing insulation was the equivalent of ~0.2 to 0.3 clo (i.e., single-layered shorts and t-shirt). Participants were instrumented while standing (~45-min) in an environmental chamber regulated at 25.0 ± 2.0°C, 40% RH and air movement of 0.2 m · s^−1^ to ensure that the subjects were thermoneutral prior to the start of testing.

Before each experimental session, the subjects sat for a baseline period of 15-min in the instrumentation chamber (25°C, 40% RH and 0.2 m · s^−1^ wind). Then they moved to an adjacent climatic chamber (40% RH and 0.2 m · s^−1^ wind), immediately started exercising for 60-min, and completed one of the four experimental treadmill exercise sessions. The sessions followed a balanced design and involved the following conditions: (1) Walking at 50% $\dot{V}O_{2\max}$ in 0°C (Walk Cold); (2) Walking at 50% $\dot{V}O_{2\max}$ in 22°C (Walk Neutral); (3) Running at 70% $\dot{V}O_{2\max}$ in 0°C (Run Cold); and (4) Running at 70% $\dot{V}O_{2\max}$ in 22°C (Run Neutral). Treadmill speed for each subject was determined individually to ensure that $\dot{V}O_{2}$ was consistently at their respective target level throughout the full 60 min. Treadmill speed for the entire exercise session averaged 6.3 ± 0.4 km · h^−1^ in Walk Cold, 6.7 ± 0.5 km · h^−1^ in Walk Neutral, 8.6 ± 0.8 km · h^−1^ in Run Cold and 8.6 ± 0.8 km · h^−1^ in Run Neutral.

### Instrumentation and measurements

Rectal temperature (T_re_) was measured using a rectal thermistor (YSI 401, Yellow Springs Instruments, USA) inserted 10 cm beyond the anal sphincter. Skin temperature was measured from six sites (face, chest, forearm, hand, thigh, and back) using thermistors (NTC DC95, Digi-Key, USA). Both core and skin temperature data were recorded by a portable data logger (SmartReader Plus 8, ACR Systems Inc., Surrey, Canada). Weighted mean skin temperature (${\bar{T}}_{\text{sk}}$) was subsequently calculated using the weighted average of the six sites (Palmes and Park, 1948):

(1) $${\bar{T}}_{\text{sk}}=0.14(T_{\text{face}})+0.19(T_{\text{chest}})+0.11(T_{\text{forearm}})+0.05(T_{\text{hand}})+0.32(T_{\text{thigh}})+0.19(T_{\text{back}})$$

A seven-point subjective scale was used to determine the subjects' thermal comfort throughout the trials (ASHRAE Standard 55, 1992). Ratings of the scale were ranging from: −3, Cold, to 0, Neutral, to 3, Hot. Subjects were required to point to their response, which was manually recorded. Heart rate (HR) was monitored using a HR monitor (T6, Suunto, Vantaa, Finland) with a continuously recording memory wirstwatch. Data was subsequently transferred and analyzed via Kubios software (KubiosHRV, Biosignal Analysis and Medical Imaging Group, Univ. Eastern Finland, Finland) for which the signal was smoothed by an artifact correction factor. Measurement for $\dot{V}O_{2}$ and respiratory quotient (RQ) were determined using an open circuit ergospirometer (Medikro 919 Ergospirometer, Medikro, Kuopio, Finland) with a gas concentration-mixing chamber located outside of the climatic chamber at thermoneutral temperature (25°C). A properly adjusted one-way Hans-Rudolph valve connected to a breathing tube was used in all trials to collect expired gases. Oxidation of CHO and fat was calculated based on stoichiometric equations (Jeukendrup and Wallis, 2005) as follows:

(2) $$\text{CHO}\left(\text{g}/\text{min}\right)=4.21\cdot \dot{V}{\text{CO}}_{2}-2.962\cdot \dot{V}{\text{O}}_{2}-0.4\cdot \text{n}$$

(3) $$\text{Fat}\left(\text{g}/\text{min}\right)=1.695\cdot \dot{V}{\text{O}}_{2}-1.701\cdot \dot{V}{\text{CO}}_{2}-1.77\cdot \text{n}$$

where n represents nitrogen excretion from protein oxidation (estimated at 135 μg · kg · min^−1^) (Romijn et al., 1993). Protein oxidation was not directly calculated as short term cold exposure does not tend to alter its contribution to energy expenditure (Vallerand and Jacobs, 1989; Haman et al., 2002). Energy expenditure was subsequently calculated for the entire 60-min of exercise based on the energy equivalent for CHO (mixture of 20% glucose and 80% glycogen; 4.07 kcal · g · min^−1^) and fat (9.75 kcal · g · min^−1^) (Jeukendrup and Wallis, 2005).

### Blood sampling and analyses

An OCRILON® polyurethane catheter (Optivia I.V 18G, Jelco, Smith's Medical, Ashford, UK), positioned in the antecubital vein before the start of the experiment and maintained throughout exercise, was used to collect blood samples in 3.5 ml vacuum-sealed serum tubes with silicon coating (BD Vacutainer® SST™ tubes, BD, New Jersey, USA) and in 3 ml K_2_EDTA whole blood tubes (BD Vacutainer® Plus Plastic K_2_EDTA tubes, BD, New Jersey, USA). Catheters were maintained using adhesive hypoallergenic, water-resistant tape (3 M™ Transpore Surgical Tape, 3M Health Care, London, Canada). Prior to centrifugation, whole blood samples were immediately analyzed for haemoglobin and haematocrit (Ac · T diff analyzer, Beckman Coulter Inc., Fullerton, CA, USA). Changes in plasma volume were calculated using haemoglobin and hematocrit changes (Dill and Costill, 1974). The blood samples were centrifuged at 3500 rpm for 10 min and were subsequently isolated in Eppendorf tubes and frozen at −80°C for future analysis.

Plasma catecholamines [epinephrine (Epi) and norepinephrine (NE)] were analyzed via a commercial ELISA kit (DRG Instruments GmbH, Germany). Coefficients of variance for intra-assay precision were 15.0% for Epi and 16.1% for NE at 2.5 ng · ml^−1^and 24.4 ng · ml^−1^ levels, respectively. Intra-assay's analytical sensitivities for Epi and NE were 0.011 and 0.044 ng · ml^−1^, respectively. Serum energy substrates [non-esterified fatty acid (NEFA), glucose, beta-hydroxybutyrate (BHB), glycerol], and serum lipids [total cholesterol (CHOL_tot_), high-density lipoprotein (HDL), and triglycerols (TG)] were analyzed by Konelab 20XTi (MedWOW, Nicosia, Cyprus). Their sensitivities of intra-assay coefficient of variances were 7.4, 2.4, 0.8, 4.6, 1.9, 2.3, and 3.4%, respectively. Low-density lipoprotein (LDL) was derived from TC and HDL values using the Friedewald equation (Friedewald et al., 1972).

### Statistical analyses

Three-way repeated measures analyses of variance (ANOVA) were used with the factors of time (levels for $\dot{V}O_{2}$, HR, RQ, fat, and CHO oxidation, core and skin temperature responses: baseline, 15, 30, 45, 60 min; levels for NEFA, glycerol, glucose, BHB, Epi, NE, and serum lipids: baseline, 30 and 60 min), ambient temperature (levels: cold and neutral), and exercise intensity (levels: walking at 50% $\dot{V}O_{2\max}$ and running at 70% $\dot{V}O_{2\max}$). A Two-Way repeated measures ANOVA with the factors of ambient temperature (levels: cold and neutral), and exercise intensity (levels: walking at 50% $\dot{V}O_{2\max}$ and running at 70% $\dot{V}O_{2\max}$) was used to determine statistical differences for total energy expenditure during the entire exercise session (60 min) as well as CHO and fat contribution. *Post-hoc* analyses were conducted using independent Tukey's HSD test when appropriate. Pearson product-moment correlations were also used to assess possible associations between catecholamines, NEFA, glycerol, glucose, BHB, and oxidation for CHO and fat. Subjective ratings from thermal comfort data was converted to ranks and was subsequently analyzed using Friedman's repeated measures ANOVA. The results are reported as mean ± SD by using *p* < 0.05 to identify statistical differences. All analyses were performed using the statistical software package Statistica 7 for Windows (StatSoft, Tulsa, OK, USA).

## Results

### Core and skin temperature and thermal comfort

**Figure 1:**
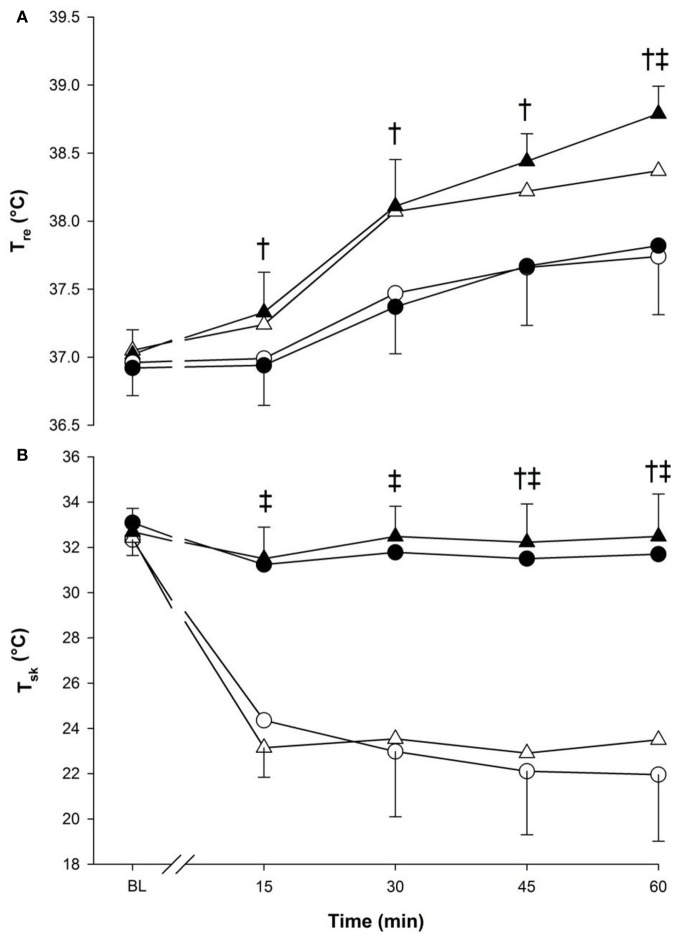
**Core (panel A) and skin (panel B) temperatures at baseline (BL) and during 60 min of exercise during walking (○ and •) and running (Δ and ▲), in cold (○ and Δ), and neutral (• and ▲) ambient temperatures.** Mean (±SD). ^†^Significant difference between walking and running (*P* < 0.05). ^‡^ Significant difference between cold and neutral (*P* < 0.001).

Core temperature (Figure 1, panel **A**) increased over time (*p* < 0.001) and was greater during running compared to walking (*p* < 0.001) (Figure 1). Moreover, an interaction between temperature and time was observed as T_re_ in cold was lower than neutral but at 60-min only (*p* < 0.001).

Skin temperature remained almost constant in neutral but dropped significantly in the cold. A three-way significant interaction was seen (*p* < 0.05) as each time point, from 15-min to the end of exercise, showed lower ${\bar{T}}_{\text{sk}}$ values in the cold compared to neutral temperatures within each exercise intensity. Walking also demonstrated lower ${\bar{T}}_{\text{sk}}$ compared to running at 45-min and 60-min (*p* < 0.05) (Figure 1, panel **B**).

The main effects of temperature and exercise both affected thermal comfort (*p* < 0.001), being lower in the cold compared to neutral and during walking compared to running. No effects were seen due to time (*p* = 0.246).

### Heart rate and oxygen consumption

HR and oxygen consumption values are presented in Table 1. HR was greater during exercise compared to baseline in all conditions (*p* < 0.001). HR was also influenced by temperature (*p* < 0.01) with lower HR values (9 ± 11 beats · min^−1^) in the cold, and by exercise intensity (*p* < 0.001) with higher values in running (29 ± 11 beats · min^−1^) compared to walking. Finally, oxygen consumption was influenced by time (*p* < 0.001) and exercise intensity (*p* < 0.001) but not by temperature (*p* = 0.925).

**Table 1:** **Oxygen uptake and heart rate during the exercise in cold and neutral environments at baseline and during exercise**.

	**Walking**		**Running**	
	**Cold**	**Neutral**	**Cold**	**Neutral**
**$\dot{V}O_{2}$ (ml · kg^−1^ · min^−1^)**
Baseline	4.5 (0.9)	3.5 (1.0)^a^	4.2 (1.1)	4.3 (1.5)^a^
15 min	25.4 (5.1)	25.0 (2.6)	36.2 (4.6)	34.8 (4.5)
30 min	24.5 (3.2)	25.2 (3.0)	37.0 (5.3)	37.3 (4.3)
45 min	24.8 (2.8)	25.3 (2.8)	37.8 (4.6)	37.0 (4.0)
60 min	24.9 (3.6)	25.4 (2.9)	37.5 (4.2)	37.0 (3.5)
**$\dot{V}O_{2}$ (%)**				
Baseline	8.9 (1.8)	6.6 (1.9)^a^	8.1 (2.0)	8.4 (2.9)^a^
15 min	48.9 (8.8)	47.4 (5.0)	69.0 (6.7)	66.3 (7.1)
30 min	46.5 (5.4)	48.1 (3.3)	69.9 (6.5)	71.6 (5.0)
45 min	47.2 (3.7)	47.7 (3.6)	70.8 (6.3)	71.6 (6.2)
60 min	47.8 (3.6)	48.3 (3.4)	70.6 (4.7)	70.5 (6.8)
**HR (beats · min^−1^)**				
Baseline	68 (16)	67 (14)^a^	70 (12)	69 (13)^a^
15 min	119 (18)	128 (7)	152 (9)	159 (11)^b^
30 min	122 (15)	133 (9)	159 (13)	170 (13)^b^
45 min	124 (12)	136 (10)	162 (14)	176 (12)^b^
60 min	126 (14)	140 (9)	163 (16)	178 (13)^b^

a Significantly lower from all other times within exercise modality (P < 0.001).

b Significantly higher in neutral temperature compared to cold within times (P < 0.001).

Values are represented as mean (±SD).

### Respiratory quotient, fuel oxidation, and energy expenditure

**Figure 2:**
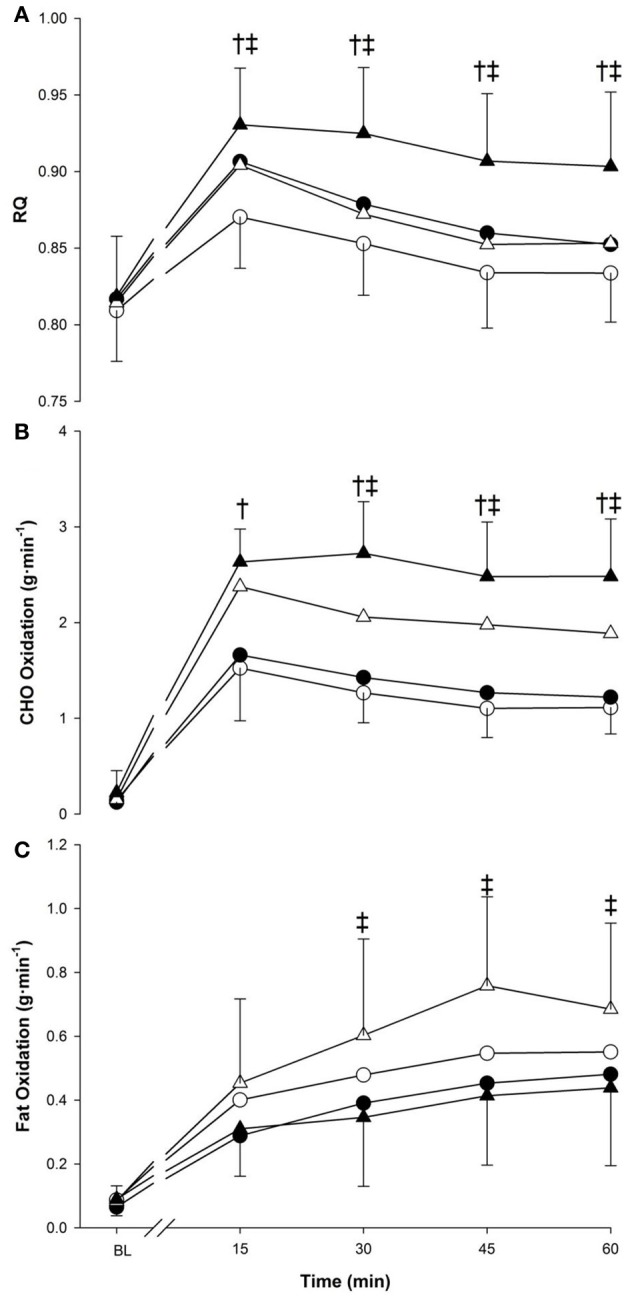
**Respiratory quotient (panel A), CHO oxidation (panel B) and fat oxidation (panel C) at baseline (BL) and during 60 min of exercise during walking (○ and •) and running (Δ and ▲), in cold (○ and Δ), and neutral (• and ▲) ambient temperatures.** Mean (±SD). ^†^Significant difference between walking and running (*P* < 0.05). ^‡^Significant difference between cold and neutral (*P* < 0.05).

RQ (Figure 2, panel A) was modulated by the main effect of time (*p* < 0.001) as it rapidly increased in the first 15-min followed by a slow decrease until the end of exercise. The main effect of temperature further influenced the response as RQ was lower in the cold (0.85 ± 0.03) compared to neutral (0.88 ± 0.03; *p* < 0.05). The main effect of exercise also indicated that RQ in running (0.88 ± 0.02) was greater than in walking (0.85 ± 0.02, *p* < 0.001). From 15 min to the end of exercise, a higher RQ response was seen in neutral temperature compared to cold (*p* < 0.01), and during running compared to walking (*p* < 0.05).

**Figure 3:**
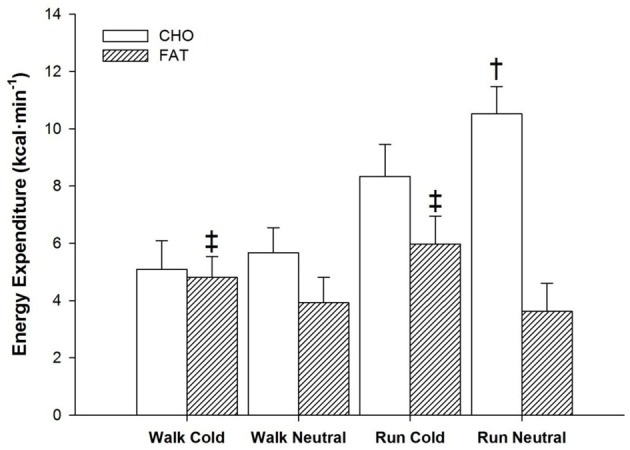
**Energy expenditure from CHO and fat sources for the entire 60 min of exercise during walking and running, in both cold and neutral ambient temperatures.** ^‡^Fat greater in cold compared to neutral across exercise (*P* < 0.05). Mean (±SD). ^†^CHO greater in Neutral compared to Cold trials (*P* < 0.05).

All main factors independently modulated the oxidation responses for both CHO and fat (*p* < 0.05) (Figure 2, panels B,C). Temperature interacted with time for CHO (*p* < 0.05) and fat (*p* < 0.005), as from 30-min to the end of exercise, CHO oxidation was greater in neutral, while fat oxidation was greater in cold. CHO oxidation was also affected by the interaction of exercise intensity and time (*p* < 0.001) with each exercise time point demonstrating greater reliance on CHO during running. This was not observed with fat oxidation (*p* = 0.259). Concerning total energy expenditure over the 60-min period, the main effect of temperature influenced both CHO and fat (*p* < 0.05) with a greater reliance of energy derived from fat in cold trials (+1.62 ± 1.99 kcal · min^−1^) and a greater reliance of CHO in neutral trials (+1.38 ± 1.09 kcal · min^−1^) (Figure 3). Exercise intensity only modulated the CHO response, which was greater during running (+4.05 ± 1.20 kcal · min^−1^, *p* < 0.001).

### Catecholamines

**Figure 4:**
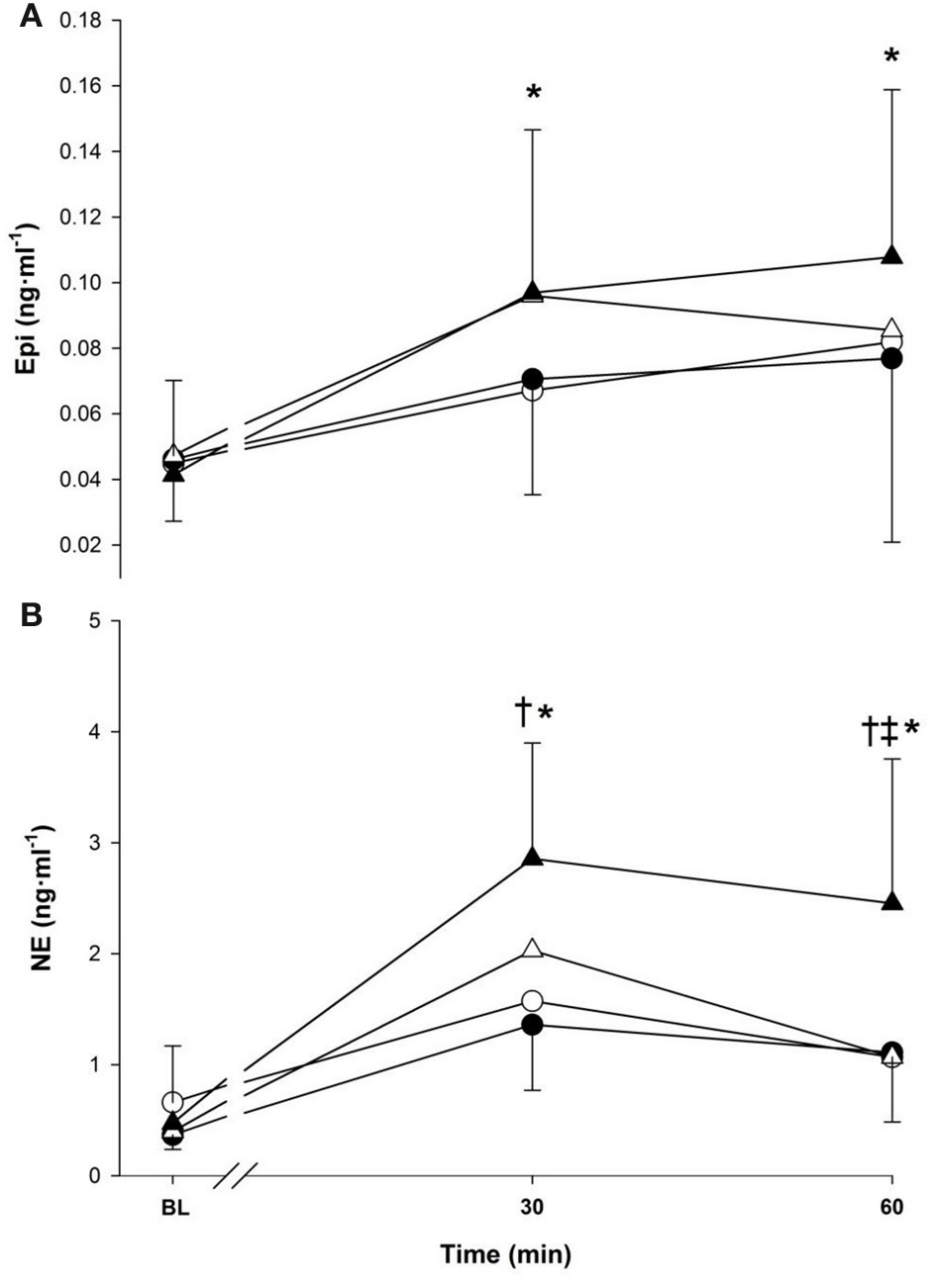
**Epinephrine (panel A), norepinephrine (panel B) at baseline (BL) and during 60-min of exercise during walking (○ and •) and running (Δ and ▲), in cold (○ and Δ), and neutral (• and ▲) ambient temperatures.** Mean (±SD). ^†^Significant difference between walking and running (*P* < 0.01). ^‡^Significant difference between cold and neutral (*P* < 0.05), ^*^Significantly greater than baseline (*P* < 0.05).

Epinephrine and NE responses are presented in Figure 4 (panels A,B). The main effect of time affected the Epi response (*p* < 0.001) with 30-min (0.08 ± 0.02 ng · ml^−1^) and 60-min (0.09 ± 0.04 ng · ml^−1^) being greater than baseline value (0.04 ± 0.02 ng · ml^−1^) (Figure 4). No effects were observed due to temperature (*p* > 0.05) or exercise intensity (*p* > 0.05).

The main effects of time, temperature and exercise intensity all modulated the NE response (*p* < 0.05), which increased over time and was greater in neutral and during running. An interaction between temperature and time was present as neutral trials had a greater response compared to cold at 60 min only (1.78 vs. 1.07 ng · ml^−1^, *p* < 0.05). Time also interacted with exercise intensity as running had a greater NE response compared to walking at 30-min (2.44 vs. 1.47 ng · ml^−1^) and 60 min (1.76 vs. 1.08 ng · ml^−1^) (*p* < 0.001).

### Lactate, NEFA, glycerol, glucose, and BHB

**Figure 5:**
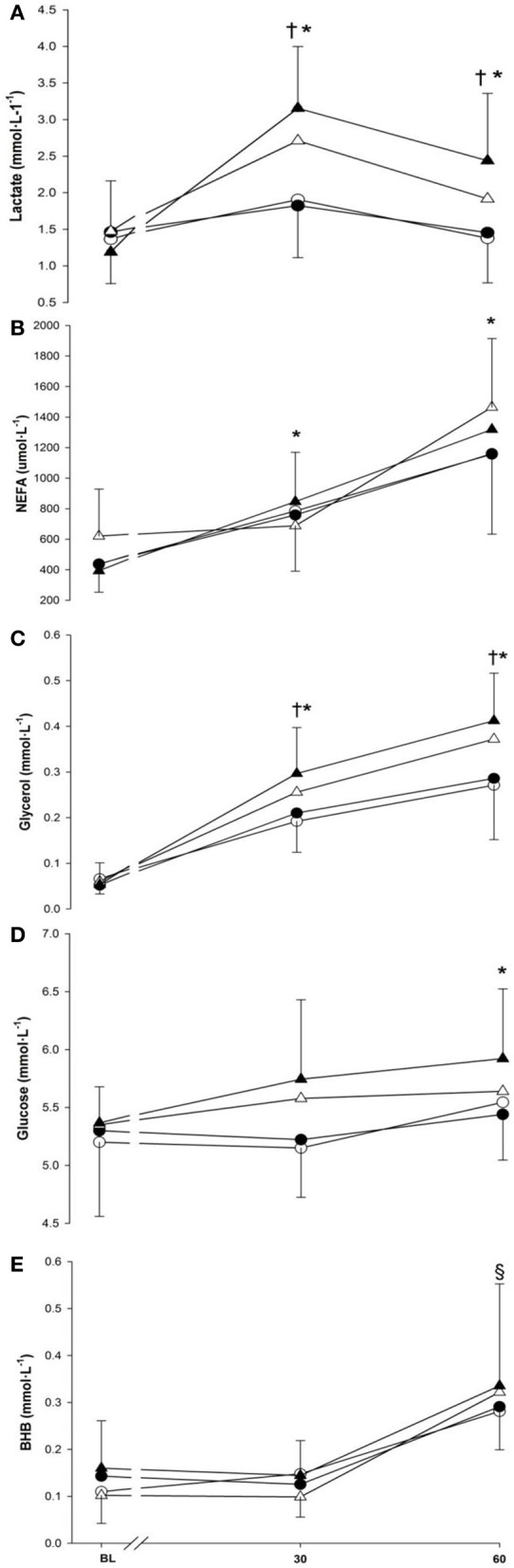
**Lactate (panel A), non-esterified fatty acids (panel B), glycerol (panel C), glucose (panel D) and beta-hydroxybutyrate (panel E) at baseline (BL) and during 60-min of exercise during walking (○ and •) and running (Δ and ▲), in cold (○ and Δ), and neutral (• and ▲) ambient temperatures.** Mean (±SD). ^†^Significant difference between walking and running (*P* < 0.01). ^*^Significantly greater than baseline (*P* < 0.05). ^§^Significantly greater than baseline and 30 min (*P* < 0.001).

Serum concentrations for lactate, NEFA, glycerol, glucose and BHB are presented in Figure 5 (panels **A**–**E**). Time and exercise intensity both modulated the lactate response (*p* < 0.001 and *p* = 0.002, respectively) with greater concentration at 30- and 60-min and during running). Nonetheless, no differences was seen between cold and neutral conditions (1.79 ± 0.48 mmol · L^−1^ vs. 1.92 ± 0.48 mmol · L^−1^; *p* = 0.439).

There was a main effect of time seen as an increase in NEFA with 30-min (769 ± 203 μmol · L^−1^) being greater than baseline (472 ± 123 μmol · L^−1^) (*p* < 0.001), and 60-min (1275 ± 230 μmol · L^−1^) being greater than both 30-min and baseline (*p* < 0.001). Neither temperature nor exercise intensity modulated the NEFA response (*p* = 0.630; *p* = 0.180). Glycerol concentration increased over time and was greater than baseline from 30-min (+0.17 ± 0.02 mmol · L^−1^) to the end of exercise (+0.27 ± 0.03 mmol · L^−1^) (*p* < 0.001). This increase continued as 60-min was also greater than 30-min (*p* < 0.001). Temperature did not have any effects on the glycerol response (*p* = 0.109). Nonetheless, a higher glycerol response was observed during running compared to walking (*p* < 0.005).

The main effects of time (*p* < 0.05) and exercise intensity (*p* < 0.001) influenced the glucose response but no change was observed due to temperature (*p* = 0.417). Glucose concentration gradually increased over time and was greater at the end of exercise (i.e., 60-min) (+0.34 ± 0.11 mmol · L^−1^, *p* < 0.05) compared to baseline. Mean glucose concentration during running was also greater compared to walking (*p* < 0.001). An increase of 0.18 ± 0.10 mmol · L^−1^ in the BHB response was seen over time as 60-min was significantly greater than baseline (*p* < 0.001). However, neither the main effects of temperature nor exercise intensity affected the BHB response (*p* = 0.531; *p* = 0.754).

### Serum lipids

Serum lipid concentrations during baseline and exercises are presented in Table 2. Temperature had no effects on any serum lipid parameters. CHOL and TG demonstrated a significant interaction between time and exercise intensity (*p* < 0.05) as baseline was lower compared to 30-min and 60-min within each exercise intensity. Triglyceride response was also greater during running compared to walking at 30-min and 60-min of exercise compared to baseline (*p* < 0.001). No significant changes were seen with LDL.

**Table 2:** **Serum lipids and plasma volume during walking and running in both cold and neutral environmental conditions at baseline and during exercise**.

	**Walking**		**Running**		
	**Cold**	**Neutral**	**Cold**	**Neutral**
**CHOL_tot_ (mmol/L)**					
Baseline	4.57 (0.73)	4.43 (0.75)^a^	4.41 (0.90)	4.51 (0.84)^a^
30 min	4.72 (0.67)	4.59 (0.93)	4.73 (0.90)	4.82 (0.82)
60 min	4.67 (0.73)	4.73 (0.97)	4.64 (0.82)	4.84 (0.85)
**HDL (mmol/L)**				
Baseline	1.60 (0.45)	1.60 (0.37)	1.58 (0.40)	1.62 (0.46)^a^
30 min	1.66 (0.46)	1.71 (0.43)	1.69 (0.40)	0.72 (0.45)
60 min	1.70 (0.52)	1.72 (0.45)	1.69 (0.42)	1.73 (0.47)
**LDL (mmol/L)**				
Baseline	2.56 (0.70)	2.52 (0.70)	2.45 (0.87)	2.49 (0.63)
30 min	2.57 (0.70)	2.48 (0.81)	2.53 (0.92)	2.59 (0.62)
60 min	2.47 (0.75)	2.57 (0.84)	2.40 (0.91)	2.56 (0.63)
**TG (mmol/L)**				
Baseline	0.88 (0.37)	0.76 (0.27)^a^	0.85 (0.36)	0.89 (0.37)^a^
30 min	1.04 (0.39)	0.92 (0.30)	1.17 (0.38)	1.13 (0.36)^b^
60 min	1.10 (0.40)	0.98 (0.20)	1.27 (0.31)	1.20 (0.34)^b^
**ΔPV (%)**				
Baseline	–	–	–	–
30 min	−2.58 (6.22)	−4.23 (8.84)	−7.95 (6.39)	−6.51 (4.67)
60 min	−1.14 (6.88)	−4.19 (8.24)	−5.62 (6.05)	−6.58 (5.23)

a Significantly lower from all other times within exercise modality (P < 0.05).

b Significantly greater during running compared to walking within times (P < 0.001).

CHOL_tot_, total cholesterol; HDL, high-density lipoprotein; LDL, low-density lipoprotein; TG, triglycerides; Δ PV, change in plasma volume. Values are represented as mean (±SD).

### Associations between energy metabolism variables

A summary of correlation coefficients for plasma catecholamines, serum energy substrates and substrate oxidation is provided in Table 3. Measured variables tended to be more related in neutral conditions than in the cold conditions both in walking and running groups.

**Table 3:** **Correlations coeffients between plasma catecholamines, serum concentrations and substrates oxidation during walking and running in both cold and neutral environmental conditions**.

	**Epi**	**NE**	**Glucose**	**NEFA**	**Glycerol**	**BHB**	**CHO_oxi_**	**Fat_oxi_**
**WALKING**								
Epi		−0.033	−0.010	+0.180	+0.149	+0.383	+0.286	+0.137
NE	+0.197		+0.079	+0.294	+0.399	−0.003	+0.299	+0.436
Glucose	+0.180	+0.206		+0.008	+0.138	+0.264	+0.042	+0.326
NEFA	+0.256	+0.251	+0.068		+0.856^*^	+0.425	+0.485	+0.638^*^
Glycerol	+0.333	+0.568^*^	+0.086	+0.892^*^		+0.468	+0.581	+0.722^*^
BHB	+0.533^*^	+0.085	+0.131	+0.493	+0.343		+0.335	+0.445
CHO_oxi_	+0.301	+0.736^*^	+0.053	+0.487	+0.766^*^	+0.379		+0.773^*^
Fat_oxi_	+0.155	+0.620^*^	+0.097	+0.695^*^	+0.836^*^	+0.212	+0.747^*^	
**RUNNING**								
Epi		+0.305	−0.111	+0.158	+0.392	+0.054	+0.476	+0.293
NE	+0.444		+0.196	+0.007	+0.440	−0.141	+0.698^*^	+0.496
Glucose	+0.165	+0.532^*^		−0.068	+0.021	−0.312	+0.232	+0.217
NEFA	+0.387	+0.428	+0.258		+0.746^*^	+0.843^*^	+0.256	+0.586^*^
Glycerol	+0.447	+0.617^*^	+0.371	+0.780^*^		+0.645^*^	+0.604^*^	+0.761^*^
BHB	+0.208	−0.003	−0.153	+0.687^*^	+0.489		+0.101	+0.492
CHO_oxi_	+0.591^*^	+0.794^*^	+0.542^*^	+0.486	+0.609^*^	−0.005		+0.530^*^
Fat_oxi_	+0.241	+0.528^*^	+0.211	+0.653^*^	+0.677^*^	+0.391	+0.400	

Upper and lower sections represent Cold and Neutral trials respectively for both Walking and Running. Epi, Epinephrine; NE, Norepinephrine; glucose; NEFA; non-esterified fatty acids; glycerol; BHB, beta-hydroxybutyrate; CHO_oxi_ (carbohydrate oxidation), and fat_oxi_ (fat oxidation).

* Significantly correlated at p < 0.01.

## Discussion

The main finding of this study was that during submaximal walking and running (i.e., whole-body exercise), a greater energetic reliance from derived fat sources occurs in the cold, despite the absence of a concurrent increase in NEFA availability or in glycerol or catecholamine concentrations. This metabolic shift was observed through a lower RQ, lower CHO oxidation, and higher fat oxidation level in cold trials with $\dot{V}O_{2}$ and core temperature maintained. This underlines the presence of a different energy selection mechanism in the cold compared to thermoneutral during exercise, which responds in a consistent manner at the present submaximal intensities of treadmill exercise.

The present results indicated that cold exposure affected the thermal, cardiovascular, and energetic responses during submaximal running and walking. Firstly, cold exposure affected ${\bar{T}}_{\text{sk}}$, which sharply decreased in the cold, and only affected *T*_re_ at 60-min during running with higher values in the neutral conditions. Secondly, lower HR values were observed throughout the entire 60-min of exercise, which is consistent with other cold exercise studies (Sink et al., 1989; Kruk et al., 1991). In the presence of skin cooling, SNS activity promotes greater peripheral vasoconstriction (Castellani et al., 1998), thereby redirecting peripheral blood flow to the core as a thermal protective mechanism. The combination of increased central blood volume and the activation of the baroreceptor reflex from vasoconstriction and stimulation of the trigeminal nerve from cold air in the face are all phenomena associated with a reduced HR in the cold (Williams and Kilgour, 1993).

Concerning the energetic response, we demonstrated a disparity between energy availability and oxidation. This was observed by a rise in reliance on fat sources in the cold with no concurrent increase in serum levels of NEFA, glycerol, glucose, BHB, or in plasma lipids. Layden et al. (2002, 2004a) observed an increase in CHO in cold environments, during cycling, with no difference in core temperature and a decrease in glycerol availability in the cold. The greater muscular stress imposed on a smaller muscle mass to perform exercise at a similar intensity and lower blood flow in cycling compared to walking or running may explain the difference in results (Hermansen and Saltin, 1969; Matsui et al., 1978). During walking at low-intensity in wet and cold environments, Weller et al. (1997a,b) demonstrated an increase in CHO reliance. In their study, low-intensity walking in the cold induced shivering thermogenesis and consequently an increase in $\dot{V}O_{2}$. It is known that shivering thermogenesis increases oxygen consumption and the use of CHO.

The increase in CHO use in the cold, seen in some studies, has suggested muscle cooling as a potential mechanism for the shift in energy requirements. Muscle cooling may reduce mechanical efficiency during exercise (Oksa et al., 2002) and, therefore, requires more oxygen to produce the same workload (Galloway and Maughan, 1997). Our protocol consisted of the maintenance of $\dot{V}O_{2}$ steadily throughout the entire exercise session to truly distinguish the metabolic effects of cold exposure without the potential interference of a change in $\dot{V}O_{2}$. We also attempted to control core temperature to avoid any decrease (through sufficient energy expenditure and heat production) as central cooling has been linked to whole-body lipolysis (Clavert et al., 1972; Hurley and Haymes, 1982). Exercise intensity can be used to predict core temperature (Nielsen, 1938; Åstrand, 2003). The increase in core temperature from exercise was delayed by the cold during running at 60 min, indicating that core temperature was not yet stabilized in the protocol. Based on the earlier response of RQ and fuel oxidation, this late difference in core temperature most likely did not affect the energetic response in any way.

Epinephrine and NE have previously been reported to respond strongly to a decrease in core temperature (Galbo et al., 1979; Weller et al., 1997a,b; Frank et al., 2002) and, to a more modest extent, with skin cooling (Weller et al., 1997a,b). We expected catecholamines to respond to the cold stimulus and modulate the oxidation response. However, our experimental protocol failed to generate significant differences in catecholamine concentrations from cold exposure and seemed to be related mostly to exercise. Importantly, increases in catecholamines related to heat stress have also been documented (Hargreaves et al., 1996). While we observed peripheral cooling in our subjects, the cold-stress may not have been severe enough to generate a cold-related increase in Epi and NE beyond the exercise stimulus. Instead, heat production from exercise most likely balanced the thermal response, and even produced low-level heat stress at 60 min in the running neutral condition, causing an increase in the NE response. Norepinephrine acts as a strong vasoconstrictive agent. We cannot discount the possibility that during running, the increase in NE and in vasoconstriction of adipose tissue beds in the thermoneutral environment would have, in part, influenced the increase in fat utilization in the cold.

We suggested that the larger muscle mass activation during walking and running, in combination with an increase in sympatho-adrenergic activity from cold exposure, would have enhanced lipolytic activity, mostly active in the upper body adipose tissue beds (Arner et al., 1990; Horowitz et al., 2000). We found little evidence from our correlations between our catecholamine data and energy substrate availability or utilization that would indicate greater lipolysis from cold exposure. This observation was noteworthy since it supports previous work that showed that an increase in substrate availability is associated with an increase in its utilization in thermoneutral [see Review by Hawley (2002)] but not in cold environments. Vallerand et al. (1999) showed evidence of an uncoupling between fatty acid availability and oxidation in the cold at rest. Layden et al. (2004a) later demonstrated the same findings in exercising subjects. In our study, the rise in fat utilization did not seem to be related to catecholamines or energy substrate availability. Therefore, other factors need to be examined to explain some of our results.

According to previous research, peripheral vascular factors may play a more central role than previously thought in energy use (Kiens et al., 1993; Layden et al., 2002). Skin temperature demonstrated the strongest response to cold exposure and peripheral capillaries may vasoconstrict to a point where skin blood flow can be almost zero. Layden et al. (2002) proposed a reduction in subcutaneous adipose tissue blood flow, which would limit NEFAs and glycerol transport during vasoconstriction. While we did not observe an actual decrease in NEFA or glycerol, reduced adipose tissue blood flow may have limited some lipolytic effects in adipocytes from cold exposure. Although skin temperature represents surface cooling, deeper tissues (i.e., subcutaneous fat and muscles) are also cooled to some extent (Oksa et al., 1995). Due to the cylindrical shape of the limbs, even a minimally extended cooling from the skin toward deeper tissues may result in high total cooled limb volume. Therefore, the role of deeper tissues may have contributed to our results. Kiens et al. (1993) suggested that lower muscle blood flow was linked to a greater affinity of working muscles to extract FFA as an energy source. The authors explained that an increase in muscle capillary density and decreased blood flow in trained vs. non-trained subjects likely induced a longer mean capillary transit time, thereby providing greater FFA extraction capability by the muscles. Although no training protocol was used in our study, therefore no change in muscle capillary density, the present vasoconstriction and peripheral tissue cooling could have possibly reduce muscle blood flow to a point, which would provide a greater FFA uptake capability. The large muscle mass activation during walking and running, in comparison to cycling, did allow some peripheral cooling (i.e., decreased skin temperature), which may have translated into some muscle cooling and reduced muscle blood flow. This potential mechanism and its effects on FFA uptake, however, need to be explored further.

Further, energy is also stored in the muscle, mostly in the form of glycogen and triglycerides. Intra-muscular cellular activity during cold exposure might explain the increase in fat reliance without the concurrent increase in plasma NEFA or glycerol. A reduced glycogenolytic rate in slightly cooled working muscle have previously been reported, even when Epi levels were similar in the control exercising muscles (Febbraio et al., 1996; Parkin et al., 1999; Starkie et al., 1999). Additionally, Romijn et al. (1993) examined the energy contribution of intramuscular triglycerides (IMTG) during submaximal exercise at 25, 65 and 85% of $\dot{V}O_{2\max}$. They found that the greatest requirement of IMTG was at 65% $\dot{V}O_{2\max}$ and contributed to ~26% of energy contribution. Our protocol used intensity levels bordering 65% $\dot{V}O_{2\max}$. For a specific workload, a decrease in availability of an energy source consequently requires the increase of another. The interaction of muscle cooling and reduced glycogenolysis would generate an energy requirement for which a compensatory reliance on another intramuscular energy source may have been possible. Since cellular processes seem to favor IMTG selection near the relative intensities used in our protocol, the observed increase in fat reliance in the cold may have indeed originated from additional IMTG contribution. Although a recent cycling study demonstrated no change in IMTG contribution to energy in the cold compared to a neutral environment (Layden et al., 2004b), more work need to be done to determine intra- and extra-cellular energy contribution to exercise in various environmental conditions.

The total energy expenditure (i.e., duration of 60 min) indicated similar results in cold and neutral but proportionally relied more on fat in the cold. This may be of interest in relation to weight loss and body composition following exercise. In a water-immersion exercise study by White et al. (2005), energy expenditure was similar for the cold and neutral water conditions, while energy intake after the cold condition was 44 and 41% higher compared to neutral and resting conditions, respectively, indicating that cold-water temperature significantly stimulated post-exercise energy intake. Interpretation of these results needs to be taken with caution as their post-exercise period was only 20 min but differences in calorie intake for similar energy expenditure may lead to changes in body weight and composition. More work is required to determine post-exercise effects in energy intake and expenditure from cold exposure.

The exercise intensities provided by our study, while offering some insights, did not cover very-low or maximal ranges. Further examination of energy and heat production, as well as fuel selection and availability is suggested as they change with exercise intensity. Also, while our study group consisted of young healthy men only lipolysis has been shown to differ in rates and tissue location between men and women (Horowitz et al., 2000). Importantly, future work should also focus on the role of IMTG and other energy sources, membrane transport and substrate cellular uptake mechanisms, in combination with localized muscle tissue blood flow and temperature.

## Conclusions

The delicate combination of thermal and exercise induced metabolic changes seems to be crucial in peripheral regulation of energy metabolism in cold environments. We observed a different fuel selection mechanism between temperate and cold environments during walking and running with greater utilization of fat as an energy substrate in the cold when core temperature and $\dot{V}O_{2}$ were maintained. Mean transit time in muscle blood flow might play a significant role in substrate uptake mechanisms during walking and running. Furthermore, consequences of a reduction in glycogenolysis with muscle cooling may impose a greater reliance on IMTG reserves to supply the energetic demand.

### Conflict of interest statement

The authors declare that the research was conducted in the absence of any commercial or financial relationships that could be construed as a potential conflict of interest.
